# From intestinal immune niches to systemic inflammation: programming cellular plasticity and immune redistribution across organs

**DOI:** 10.3389/fimmu.2026.1888071

**Published:** 2026-09-15

**Authors:** Bohang Zeng, Qiuling Xiong, Cai He, Lirui Sun, Yan Wang, Shuai Zhang, Jiaying Xin, Xuebo Liu, Jianbo Cheng, Yutong Song

**Affiliations:** 1 College of Food Engineering, Harbin University of Commerce, Harbin, China; 2 College of Food Science and Engineering, Northwest A&F University, Yangling, China; 3 Key Laboratory for Food Science & Engineering, Harbin University of Commerce, Harbin, China; 4 Heilongjiang Feihe Dairy Co., Ltd, Qiqihar, China

**Keywords:** gut microbiota, gut-organ axis, immune cell migration, mucosal immunology, systemic inflammation

## Abstract

Immune-mediated inflammatory diseases have traditionally been investigated within organ-specific frameworks. Although increasing evidence has revealed extensive communication along individual gut-organ axes, these interactions are often described through separate mechanisms, including microbial dysbiosis, barrier disruption, soluble mediators, and immune-cell redistribution. A central challenge remains to determine how intestinal immune states are established and how they are translated into organ-specific inflammatory responses. In this review, we propose that the intestine functions as a dynamic immune programming niche for mobile immunity, where microbial, epithelial, and stromal signals collectively shape immune-cell states before their systemic dissemination. Through this niche-dependent education, immune cells acquire distinct phenotypic and migratory programs, while retaining sufficient plasticity to be further reconfigured within peripheral tissues. By integrating evidence across the gut-liver, gut-joint, gut-skin, gut-brain, and other organ axes, we establish a framework in which intestinal immune niches are followed by systemic trafficking, tissue-specific recruitment, and local functional reprogramming. This perspective shifts the focus of gut-organ communication from isolated organ interactions toward a directional process involving immune-cell generation, trafficking, and tissue-specific adaptation. This perspective reframes gut-organ communication as gut-driven immune redistribution rather than isolated organ-axis interactions, highlights intestinal immune programming and immune-cell trafficking as potential targets for systemic immune intervention.

## 1 Introduction

Immune-mediated inflammatory diseases have traditionally been investigated within individual organs, despite increasing evidence that immune disturbances in one tissue can influence inflammatory responses elsewhere. Clinical observations increasingly indicate that intestinal perturbations are associated with immune dysregulation in distant tissues, suggesting that the intestine participates in bidirectional communication with peripheral organs. Inflammatory bowel disease affects not only the gastrointestinal tract but also the joints, skin, eyes, hepatobiliary system, and lungs, resulting in diverse extraintestinal manifestations. These observations suggest that intestinal inflammation can influence distant organs through sustained immunological communication rather than isolated inflammatory spillover (1–3). Accordingly, the ‘gut-organ axis’ has emerged as an important framework for understanding multiorgan inflammation, referring here to bidirectional immunological and molecular interactions between the intestine and specific peripheral organs. Most previous studies, however, have focused on a single disease or an individual organ axis. Dysbiosis, barrier disruption, circulating inflammatory mediators, and immune cell migration are often presented as parallel mechanisms. Few studies have systematically evaluated their relative contributions or clarified when immune-cell trafficking represents a causal link rather than an associated feature of organ-selective pathology. Therefore, understanding gut-organ communication requires not only identifying shared inflammatory signals but also determining how intestinally conditioned immune cells acquire migratory capacity, reach specific tissues, and undergo functional reactivation after arrival.

To understand how intestinal signals influence distant organs, gut–organ communication can be considered through three complementary processes: dissemination of soluble mediators, translocation of microbial or antigenic components, and trafficking of immune cells. The first involves the systemic dissemination of soluble mediators. Microbe-associated molecular patterns, microbial metabolites, and host-derived cytokines, hormones, and metabolic signals enter the portal or systemic circulation. They may then act directly on parenchymal cells, vascular endothelial cells, and immune cells in distant tissues (4, 5). The second route involves barrier disruption-mediated exposure to microbial or antigenic materials. Viable bacteria, fungi, microbial structural components, and other antigenic substances cross the damaged intestinal mucosal barrier. They subsequently reach extraintestinal tissues through the portal vein, lymphatic system, or systemic circulation (5–7). The third involves immune-cell trafficking, whereby intestinally conditioned immune cells act as mobile carriers of local immune information, through antigenic stimulation, metabolic cues, and functional reprogramming, intestinal immune cells acquire phenotypes that enable their migration and subsequent regulation of distant tissues. These cells may subsequently traffic toward distant tissues, where their functional consequences are determined by local antigen presentation, chemokine gradients, and tissue-specific inflammatory environments (8, 9).

These processes are not independent pathways but sequential and interacting events that shape the transition from intestinal perturbation to distal immune responses. Impaired intestinal barrier function increases exposure to microbial components, which can influence both local immune programming and systemic inflammatory responses. Microbial molecules and metabolites further shape immune cell differentiation, functional states, and migratory potential within the gut. They can also act on immune cells, vascular endothelial cells, and stromal cells in distant tissues, thereby reshaping the local inflammatory state and cellular recruitment environment (4–7). Following intestinal immune conditioning, selected immune-cell populations acquire migratory competence and can enter the circulation under the control of chemokine and adhesion programs. These cells may retain antigen experience, clonal characteristics, and effector or regulatory properties that influence their function after reaching distant tissues (8, 9). Thus, soluble signals, microbial or antigenic translocation, and immune cell migration may contribute, respectively, to inflammatory initiation, remodeling of the target-tissue microenvironment, and transfer of antigen-specific immune programs. Together, these processes explain how intestinal perturbations can be translated into organ-specific immune responses through both systemic signals and the redistribution of programmed immune cells.

Based on this framework, we propose that gut-organ immune communication can be understood as a sequential process involving intestinal immune programming, systemic trafficking of conditioned immune cells, and distal tissue selection and reactivation. We evaluate evidence for this model across the gut-liver, gut-joint, gut-skin, and gut-brain axes, with particular emphasis on whether immune cells originate from the intestine, how they migrate, and how their functions are modified after entering distant tissues. We integrate evidence regarding the potential intestinal origin of migratory immune cells and the molecular cues that govern their mobilization. We also examine the chemokine and adhesion pathways that direct tissue localization, together with evidence of immune-cell reactivation and functional remodeling in distant organs. By comparing evidence across organ systems, we aim to distinguish associations from experimentally supported directional trafficking and causal involvement. We further discuss therapeutic strategies targeting different stages of this process, including intestinal immune programming, immune-cell migration, and distal tissue activation. It may also explain why similar intestinal abnormalities lead to divergent organ-specific outcomes and facilitate the identification of mechanistic targets for patient stratification and cross-organ intervention.

## 2 Intestinal immune programming

### 2.1 Epithelial-microbial interfaces shape immune programming

In this review, intestinal immune programming refers to the process by which epithelial signals, microbial-derived cues, and antigen-presenting networks shape the phenotype, function, and migratory potential of immune cells within the gut. The intestinal barrier represents the initial interface of immune programming, where microbial products, dietary antigens, and inflammatory mediators are sensed and integrated by mucosal immune cells (10). This barrier is a multilayered interface composed of epithelial cells, tight junctions, mucus, antimicrobial molecules, immune mediators, and epithelial repair pathways (11). Tight junctions regulate paracellular permeability through pore and leak pathways, and their disruption alters molecular flux across the intestinal epithelium during inflammatory disease (12). Bel et al. demonstrated in Salmonella-infected mice, Paneth cells, and models carrying *Atg16l1* risk variants that bacteria-induced endoplasmic reticulum stress activates secretory autophagy. This pathway preserves antimicrobial defense by enabling extracellular lysozyme release despite secretory defects. It is regulated by mucosal innate immune signals and restricts bacterial dissemination beyond the intestine (13). Goblet cells secrete mucins to form a mucus layer overlying the intestinal epithelium. Beyond physical separation, this mucus interface also influences the quality of microbial signals presented to immune cells. This layer limits microbial contact with epithelial cells while also contributing to the regulation of mucosal immunity (14).

Soluble signals derived from the intestinal epithelium further define the thresholds for T-cell differentiation and function. Harrison et al. examined epithelial-derived IL-18, IL-18R1-deficient CD4^+^ T cells, and experimental colitis models. They found that IL-18, constitutively secreted by the epithelium under homeostatic conditions, acts directly on lamina propria CD4^+^ T cells. This signal limits colonic Th17 differentiation, partly by antagonizing IL-1R1 signaling. Although IL-18R1 signaling is not required for Treg generation, it enhances the expression of effector molecules needed for Foxp3^+^ Treg cells to control intestinal inflammation (15). These findings indicate that epithelial cells not only restrict antigen entry but also directly set the activation thresholds and functional direction of effector and regulatory T-cell responses, thereby shaping downstream immune-cell differentiation.

Microbial metabolites further translate microbiota-derived functions into signals that determine T-cell fate. Hang et al. first screened a library of bile acid metabolites. They then combined *in vitro* polarization of naive CD4^+^ T cells with receptor-binding assays, reporter assays, and *in vivo* administration in mice. This approach identified two metabolites with opposing immunomodulatory effects, 3-oxoLCA and isoalloLCA. 3-oxoLCA directly binds RORγt and suppresses its transcriptional activity, thereby limiting Th17 differentiation. In contrast, isoalloLCA promotes Treg differentiation by increasing mitochondrial reactive oxygen species and Foxp3 expression (16). Thus, microbial metabolism does not merely alter inflammatory intensity but can reshape the balance between regulatory and pathogenic T-cell programs.

In summary, the intestinal epithelium controls immune stimulation through structural compartmentalization, mucosal secretion, and selective antigen transport, thereby shaping the type and intensity of immune responses generated in the underlying mucosa. Epithelial-derived cytokines and microbial metabolites further define the threshold for Treg–Th17 differentiation. Thus, this interface functions not only as a physical barrier but also as an immune-instruction platform that shapes subsequent immune programming within the lamina propria.

### 2.2 Lamina propria immune networks establish migratory immune programs

The lamina propria functions as a central immune programming niche where epithelial and microbial inputs are translated into cellular phenotypes with distinct effector and migratory properties (17).

Lamina propria myeloid cells direct T-cell differentiation through distinct cytokine combinations. Denning et al. separately isolated CD11b^+^F4/80^+^CD11c^−^ macrophages and CD11b^+^ dendritic cells from the lamina propria and cocultured them with naive CD4^+^ T cells. Even after Toll-like receptor stimulation, macrophages predominantly produced suppressive mediators such as IL-10. They promoted Foxp3^+^ Treg differentiation through IL-10, retinoic acid, and exogenous TGF-β. In contrast, lamina propria dendritic cells more readily induced IL-17 production. When the two cell types were cocultured, macrophages also suppressed dendritic cell-driven IL-17 responses (18). Thus, intestinal T-cell fate is not determined by a single antigen-presenting population but emerges from interactions among myeloid subsets that establish local inflammatory or regulatory programs.

ILCs influence T-cell responses primarily by regulating the local immune microenvironment and through MHC-II-dependent interactions. This mechanism differs from the clonotypic antigen recognition and homing-receptor imprinting that operate intrinsically in T cells. Hepworth et al. combined genetic or antibody-mediated ILC depletion with transcriptomic analysis, antigen-processing and presentation assays, and mice lacking MHC-II specifically in RORγt^+^ ILCs. They found that ILC3s express MHC-II and process and present commensal antigens, but lack the typical costimulatory activity required to promote conventional T-cell expansion. In contrast, selective deletion of MHC-II in ILC3s caused uncontrolled commensal-dependent CD4^+^ T-cell responses and spontaneous intestinal inflammation (19). ILC3s therefore act as a local checkpoint that restrains excessive expansion of commensal-specific T cells and regulates the threshold of T-cell activation through an MHC-II-dependent mechanism.

Dendritic cells provide the most direct link among antigen sensing, T-cell differentiation, and migratory imprinting. Dzutsev et al. cocultured OVA-specific OT-I CD8^+^ T cells with CD11c^+^ antigen-presenting cells derived from either the small intestine or colon. The findings were further validated by flow cytometry, ALDEFLUOR assays, RT-PCR, and adoptive-transfer homing experiments. Small-intestinal antigen-presenting cells induced the expression of both α4β7 and CCR9 on T cells. By contrast, colonic antigen-presenting cells predominantly induced α4β7 and had a weaker effect on CCR9 expression, highlighting that intestinal homing imprinting is region-specific rather than governed by a universal α4β7–CCR9 program. Colonic antigen-presenting cells also expressed lower levels of retinoic acid-synthesizing enzymes than their small-intestinal counterparts. These findings support a model in which low retinoic acid levels are sufficient to induce α4β7 but insufficient to fully induce CCR9, thereby generating an α4β7^+^CCR9^low colonic-homing phenotype. Subsequent homing experiments using β7- or CCR9-deficient cells showed that β7 deficiency markedly impaired CD8^+^ T-cell entry into the colonic lamina propria, whereas CCR9 deficiency did not affect colonic homing (20). Other adhesion molecules, including αEβ7 (CD103) and β2 integrins such as LFA-1, further contribute to immune-cell retention and local positioning within intestinal tissues, highlighting that α4β7–MAdCAM-1 represents one component of a broader trafficking network.

These regional differences do not imply that the small intestine and colon use entirely separate adhesion systems. Berlin et al. performed *in vitro* adhesion assays using purified MAdCAM-1, MAdCAM-1-transfected cells, and integrin-blocking antibodies. They showed that α4β7 binds MAdCAM-1 with high specificity and efficiency. Blocking either α4 or β7 markedly inhibited lymphocyte adhesion, thereby establishing the α4β7–MAdCAM-1 interaction as a common adhesion axis for lymphocyte entry into the intestinal mucosa and associated lymphoid tissues (21). Intestinal segment selectivity is therefore better described as a shared adhesive scaffold overlaid by region-specific antigen presentation and chemotactic programs that imprint future tissue distribution. Colonic homing, by contrast, retains α4β7-mediated adhesion while incorporating GPR15-dependent directional cues (22).

Collectively, the lamina propria should be viewed as a functional programming niche rather than a static immune compartment. Dendritic cells define antigen specificity and migratory potential, macrophages set the inflammatory tone, and ILCs stabilize or remodel the epithelial-stromal environment. The balance among these modules determines whether gut-conditioned immune cells acquire regulatory, inflammatory, or migratory phenotypes capable of influencing distal tissues. The intestinal immune programming mechanisms discussed above are schematically depicted in **Figure 1**.

**Figure 1:**
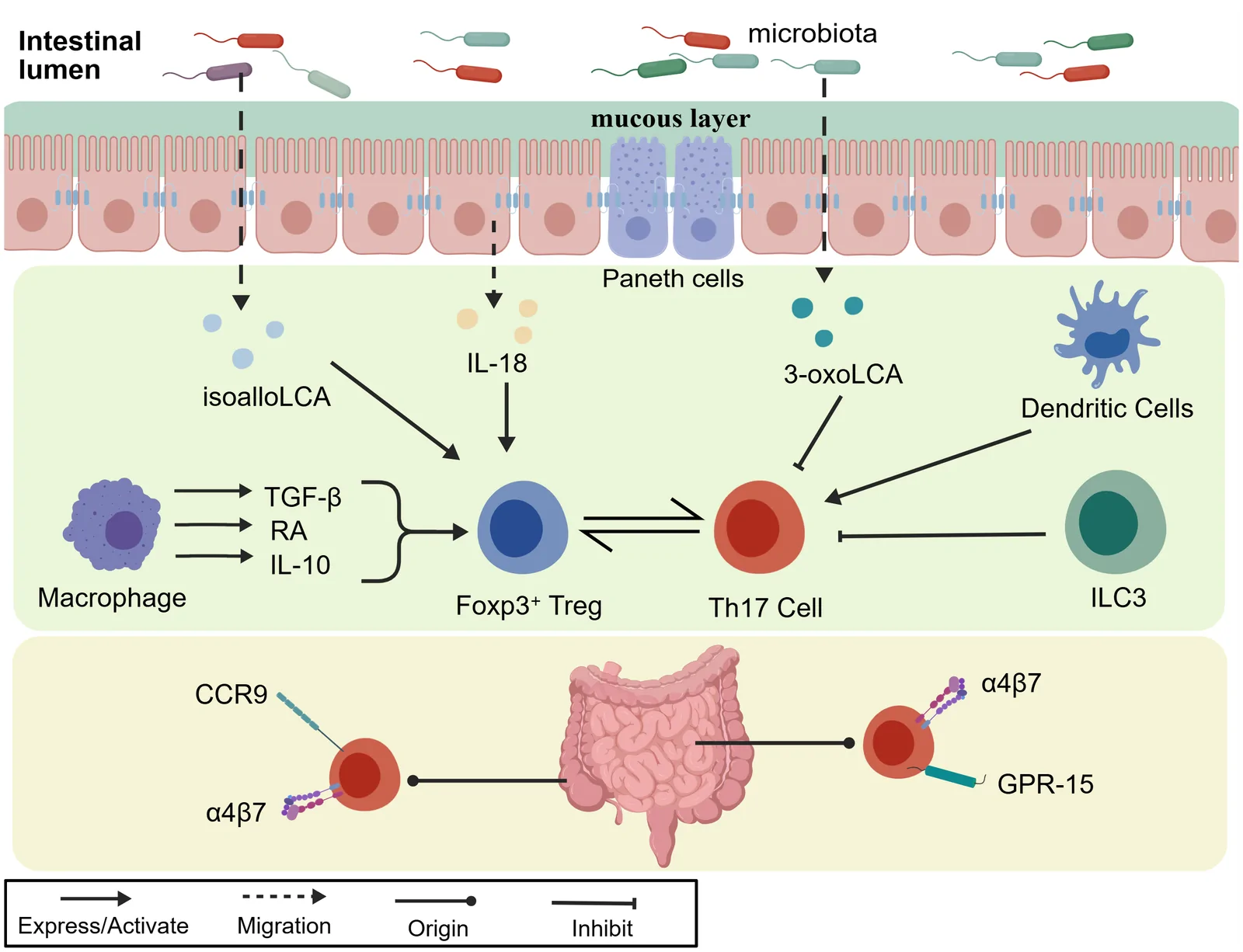
Intestinal immune niches program mobile immune states before systemic redistribution. Epithelial barrier status, mucus organization, microbial antigens, and microbiota-derived metabolites initiate immune education. Dendritic cells, macrophages, and innate lymphoid cells in the lamina propria translate these inputs into tolerogenic, repair-associated, or inflammatory programs. Peyer’s patches and mesenteric lymph nodes further coordinate antigen capture, lymphocyte activation, and α4β7/CCR9-dependent gut-homing imprinting. Together, these events convert local mucosal information into mobile immune phenotypes with systemic migratory potential. Created with BioGDP.com (127).

## 3 Gut-derived immune-cell trafficking and tissue-specific reprogramming across organs

### 3.1 Gut–liver immune communication

#### 3.1.1 Intestinal imprinting of hepatic immunity

Intestinal inflammation can reshape hepatic immune surveillance before overt liver injury develops. Remodeling of intrahepatic antigen-presenting cell populations represents an early consequence of gut-derived inflammatory signals and creates a permissive environment for subsequent hepatic inflammation. Using models of chronic T-cell transfer colitis, IL-10-deficient colitis, and acute DSS-induced colitis, Mathies et al. found that intestinal inflammation reduced the number of hepatic plasmacytoid dendritic cells while increasing pro-inflammatory macrophages and conventional dendritic cells. The latter two populations promoted CD4^+^ T-cell proliferation and induced Th1 differentiation. Notably, DSS still induced severe colitis in germ-free mice, but the hepatic accumulation of pro-inflammatory macrophages and dendritic cells was markedly reduced. Moreover, colitis-conditioned hepatic myeloid cells exacerbated subsequent Fas-mediated hepatitis. These findings indicate that hepatic immune remodeling is not merely a passive reflection of systemic inflammation. Rather, it requires intestinal microbial signals and barrier-dependent exposure to microbial components. When the intestinal barrier is compromised, increased exposure to microbe-associated molecules may alter the balance of hepatic antigen-presenting cells and increase susceptibility to inflammatory liver injury (23).

Microbiota-dependent barrier disruption further links intestinal abnormalities to intrahepatic effector T-cell responses. In a PSC-related model, Nakamoto et al. colonized mice with patient-derived microbiota. They found that specific pathobionts induced intestinal epithelial barrier dysfunction and bacterial translocation, accompanied by enhanced hepatic Th17 responses (24). This study supports a causal sequence involving dysbiosis, barrier disruption, and hepatic immune activation. However, such phenotypic and interventional evidence alone does not establish the directional migration of gut-derived T cells into the liver. Spatially resolved and direction-specific cell-tracing studies are therefore required.

#### 3.1.2 Gut-derived immune-cell trafficking to the liver

Direct cell tracing provides directional evidence that gut-derived immune cells can migrate from the intestine to the liver. In DSS-induced and T-cell transfer colitis models, Mathies et al. observed bacterial translocation, increased hepatic Th17 cells, and inflammatory-cell infiltration. Intestine-restricted Kaede photoconversion further demonstrated that a subset of labeled immune cells migrated from the inflamed intestine to the liver (23). However, migration did not necessarily result in tissue injury. DSS-induced colitis increased hepatic Treg cells without causing overt liver pathology. By contrast, hepatic Th17 accumulation and liver injury became markedly aggravated in Treg-deficient transfer colitis models or after Treg depletion in DEREG mice. These findings suggest that gut-derived immune-cell migration represents the output of an intestinal immune niche that programs cellular identity before redistribution to the liver. Its pathogenic consequences, however, depend on hepatic immune context, including local tolerance mechanisms and the balance between effector T cells and Treg cells.

Models of alcohol-related liver disease have further integrated migration direction, antigen specificity, and pathogenic activity into a single chain of evidence. Yang et al. showed that ethanol-induced intestinal fungal dysbiosis aggravated hepatitis through β-glucan translocation and CLEC7A signaling in Kupffer cells (25). Building on this observation, Zeng et al. demonstrated that ethanol exposure increased intestinal *Candida albicans*-reactive Th17 cells. Kaede tracing further confirmed that these cells migrated from the intestine or mesenteric lymph nodes to the liver. Nystatin treatment reduced hepatic *C. albicans*-specific Th17 cells and alleviated liver pathology. Conversely, disease was aggravated in *C. albicans*-reactive TCR-transgenic mice and after the adoptive transfer of antigen-specific T cells or polyclonal T cells primed with *C. albicans in vitro*. This pathogenic effect required IL-17RA signaling in Kupffer cells (26). Thus, this study provided not only the cellular origin and direction of migration but also antigen specificity, effector sufficiency, and the requirement for a local receptor pathway.

#### 3.1.3 Hepatic reactivation of gut-derived immunity

Entry into the liver does not necessarily mean that migrating cells have established a sustained pathogenic effect. Disease development usually requires several sequential steps. These include vascular recruitment and transendothelial migration, tissue retention and survival, and cytokine-driven effector amplification or local reactivation by cognate antigen.

The entry of gut-derived T cells, or T cells with a mucosal-homing phenotype, first requires inflamed hepatic vessels to establish a recruitment program that matches the receptors expressed by these cells. Hillan et al. found that MAdCAM-1 was aberrantly expressed in several liver diseases characterized by portal tract inflammation. This finding suggests that MAdCAM-1 is not specific to PSC but instead reflects the acquisition of a mucosal-like endothelial phenotype by the inflamed liver (27). Using human liver tissues and *in vitro* flow-based adhesion assays, Grant et al. further showed that portal venous and sinusoidal endothelial cells from chronically inflamed livers supported the adhesion of α4β7^+^ lymphocytes. Blocking either MAdCAM-1 or α4β7 inhibited this process (28). Ando et al. subsequently demonstrated that inflammatory cytokines induced MAdCAM-1 expression in hepatic endothelial cells and enhanced α4β7-dependent lymphocyte–endothelial adhesion (29). In a Western diet-induced steatohepatitis model, antibiotic treatment reduced MAdCAM-1 levels in both the colon and liver. Blockade of α4β7 decreased the accumulation of α4β7^+^ CD4^+^ T cells in both tissues and simultaneously improved inflammation, fibrosis, and metabolic abnormalities (30). Together, these findings indicate that the α4β7–MAdCAM-1 axis not only mediates adhesion but may also contribute to disease-associated T-cell recruitment into the liver in selected inflammatory contexts.

Complete hepatic recruitment also requires chemokine-guided positioning and integrin activation. Adhesion molecule expression alone is insufficient. Eksteen et al. detected CCL25 in hepatic endothelial cells from patients with PSC and identified a CCR9^+^α4β7^+^ population among liver-infiltrating lymphocytes. This mechanism appears to represent a subset-specific recruitment pathway rather than a universal homing program. Functional experiments showed that CCL25 promoted the interaction of these cells with MAdCAM-1 (31). Under shear-flow conditions, Miles et al. further demonstrated that CCL25 and CCL28 enhanced α4β7 adhesive activity through G protein-dependent inside-out signaling (32). Liaskou et al. also found that, under TNF-mediated inflammatory conditions, VAP-1 substrates such as methylamine induced functional MAdCAM-1 expression through VAP-1 amine oxidase activity. This process enhanced the adhesion of mucosal effector lymphocytes to hepatic vessels (33). The entry of gut-derived T cells into the liver can therefore be described as a continuous sequence of chemokine-guided positioning, integrin activation, stable adhesion, and transendothelial migration. Portal metabolic cues and inflammatory cytokines jointly establish a hepatic microenvironment that favors cellular recruitment.

After transendothelial migration, the sustained accumulation of T cells in the liver depends on retention and survival signals provided by local tissue niches. Afford et al. found that biliary epithelial cells expressed VCAM-1 in chronic liver disease. The interaction of VCAM-1 with α4β1 on T cells not only mediated adhesion but also delivered anti-apoptotic signals. This interaction reduced effector T-cell apoptosis and prolonged their residence within portal tracts (34).

Whether liver-infiltrating T cells subsequently cause tissue injury depends on local cytokine amplification and antigen-specific reactivation. Jeffery et al. found that LPS and Th1/Th17-associated inflammatory cytokines stimulated human biliary epithelial cells to release CCL20, IL-1β, and IL-6. These mediators promoted Th17-cell chemotaxis, phenotype maintenance, and local expansion. In turn, Th17-associated cytokines promoted biliary epithelial cell proliferation, thereby establishing a bidirectional positive-feedback loop (35). This mechanism may explain how the effects of migrating cells are amplified within the liver.

Organ-specific pathology may also arise when migrating T cells recognize cognate antigens in the liver and undergo local reactivation. Seidel et al. established an experimental system in which intestinal and biliary epithelial cells expressed the same model antigen. CD8^+^ T cells primed by cognate antigen in GALT acquired both effector activity and the capacity to enter the liver. However, the migrating cells induced overt cholangitis only when the same antigen was expressed by biliary epithelial cells. DSS-induced colitis further enhanced this T-cell-dependent biliary injury by increasing portal inflammatory mediators and activating intrahepatic dendritic cells (36). This study indicates that gut-derived immune cells can retain their original antigen specificity after activation. Following migration to the liver, they can be reactivated by the same antigen, thereby providing a mechanistic explanation for how identical intestinal immune programs can generate distinct organ-specific outcomes after migration.

Together, current evidence supports a multistep model in which intestinal inflammation reshapes hepatic immune susceptibility, enables recruitment of gut-derived immune cells, and permits their local reactivation within the liver. Microbial dysbiosis and barrier dysfunction reshape hepatic immune susceptibility, whereas the migration of gut-derived T cells provides a cellular basis for cross-organ immune communication. Migration alone, however, is insufficient to cause overt liver disease. Its pathological consequences are jointly determined by hepatic recruitment, tissue retention, cytokine-mediated amplification, and local antigen-dependent reactivation.

### 3.2 Gut–joint immune communication

#### 3.2.1 Intestinal imprinting of joint inflammation

RA-associated microbiota may establish pro-inflammatory mucosal immune programs during the preclinical phase, before the clinical onset of arthritis. Maeda et al. colonized germ-free SKG mice with *Prevotella copri*-enriched microbiota derived from patients with early RA. The recipient mice showed increased colonic Th17 cells and enhanced IL-17 responses to the autoantigen RPL23A in the colon and regional lymph nodes. They also developed more severe arthritis after zymosan challenge. In addition, *P. copri*-stimulated dendritic cells induced naive SKG T cells to acquire pro-inflammatory reactivity against RPL23A. These cells rapidly triggered arthritis after adoptive transfer (37). Luo et al. further examined individuals with preclinical RA and found that their gut microbiota and serum metabolomic profiles were altered before disease onset. Transfer of microbiota from these donors into antibiotic-pretreated mice increased intestinal permeability and expanded Th17 cells in Peyer’s patches and mesenteric lymph nodes. The recipient mice subsequently developed more severe collagen-induced arthritis (CIA) (38). Collectively, these studies support the intestine as an upstream immune compartment that shapes arthritic susceptibility through microbial-dependent barrier disruption and T-cell polarization. These findings suggest that microbial risk is context-dependent and requires interaction between microbial traits and host immune susceptibility.

The arthritogenic potential of the microbiota also depends on interactions between specific microbial components and host immune thresholds. Maeda et al. found that colonization with *Palleniella intestinalis* increased intestinal permeability and selectively activated colonic CD11b^+^CD11c^+^ myeloid cells. Outer-membrane vesicles derived from either *P. intestinalis* or *Segatella copri* induced bone marrow-derived dendritic cells to produce IL-6 and enhanced Th17 differentiation. Transfer of these vesicles or bacterially preconditioned dendritic cells was sufficient to increase arthritis incidence in relatively resistant mice (39). Sendo et al. compared microbiota from several RA donors in germ-free PTPN2 wild-type and haploinsufficient SKG mice. Only a subset of donor microbiota aggravated arthritis in the setting of PTPN2 haploinsufficiency. Monocolonization with *P. copri* similarly expanded joint CD4^+^RORγt^+^Foxp3^−^ T cells and exacerbated disease (40). Thus, microbial pathogenicity is not determined by the abundance of a single genus. Rather, it is amplified by interactions between microbial effects and host genetic susceptibility.

Microbial metabolites may also regulate arthritis remotely through humoral pathways and represent a complementary route of gut-joint communication alongside cellular trafficking. In CIA, Seymour et al. found that bacterial tryptophanase and dietary tryptophan jointly maintained luminal indole production. Microbiota depletion, dietary tryptophan restriction, or colonization with indole-deficient bacteria reduced arthritis. In contrast, indole supplementation restored disease and increased IL-6, TNF, IL-1β, RORγt^+^CD4^+^ T cells, collagen-specific IL-17 responses, and pro-inflammatory antibody features (41). By contrast, Wu et al. reported reduced activity of the indole pathway in individuals with preclinical RA and established RA. Supplementation with indole-3-lactic acid or indole-3-acetic acid expanded Treg cells, restored tight-junction integrity, and alleviated CIA through AhR signaling. These protective effects were reversed by AhR antagonism (42). Collectively, these studies establish causal links between intestinal microbes, their metabolites, and arthritis. They also provide an upstream basis for testing whether gut-derived immune cells directly reach the joints.

#### 3.2.2 Gut-derived immune-cell trafficking to the joint

Local photoconversion of the small intestine first placed intestinal barrier disruption, gut-derived CD4^+^ T-cell migration, and joint injury within the same experimental framework. In a longitudinal CIA model, Tajik et al. found that increased serum zonulin, reduced tight-junction proteins, and elevated small-intestinal permeability all preceded clinically apparent arthritis. The investigators selectively photoconverted the small intestine of Kaede mice and detected Kaede-red^+^ cells in lymph nodes, the spleen, and synovial tissue 4 days later. Among these sites, the synovium contained the highest proportion of small intestine-derived CD4^+^ T cells. Short-term treatment with the zonulin antagonist larazotide before disease onset restored barrier integrity, markedly reduced gut-derived labeled cells in the synovium, and concurrently alleviated synovitis, osteoclast formation, and bone erosion (43). This study provided direct evidence that small-intestinal immune cells migrate to the synovium during the initiation of arthritis. It also showed that barrier integrity determines the extent of this cellular dissemination.

SpA-related models further localized the migratory source to colonic intraepithelial T cells and showed that these cells retain pro-inflammatory activity. Lefferts et al. labeled distal colonic intraepithelial lymphocytes *in situ* in KikGR mice. KikRed^+^ T cells were subsequently detected at the Achilles tendon enthesis and in its draining lymph nodes. Migrating CD4^+^ T cells were enriched for IL-17 production. In TNF^ΔARE/+^ mice, these cells also retained a strong capacity to produce TNF. Transfer of colonic intraepithelial lymphocytes from TNF^ΔARE/+^ mice into Rag1^−/−^ recipients aggravated arthritis. Conversely, broad-spectrum antibiotics reduced the recruitment of colonic intraepithelial lymphocytes and decreased gut-to-joint migration, peripheral IL-17A/TNF responses, and joint pathology (44). This study established a continuous mechanistic link among colonic origin, migration to the enthesis, effector-cytokine production, and disease aggravation.

#### 3.2.3 Joint reactivation of gut-derived immunity

Gut-derived T cells may first enter systemic lymphoid organs and amplify arthritis through germinal-center and autoantibody responses. Direct entry into the synovium is not required for all pathogenic cells. In K/BxN mice, Wu et al. found that germ-free conditions markedly reduced intestinal Th17 cells, autoantibody production, and arthritis. Colonization with segmented filamentous bacteria restored these phenotypes, indicating that microbiota-induced intestinal T-cell responses act upstream of disease (45). Block et al. subsequently used genetic interventions targeting Bcl6 and IL-17. They found that the microbiota-dependent promotion of arthritis required the Tfh program rather than IL-17 itself. Bcl6 deficiency suppressed germinal-center formation, autoantibody production, and arthritis, whereas inhibition of Th17 generation did not provide comparable protection (46). These results position intestinal Th17 expansion as an upstream immune state, while identifying Tfh–B-cell interactions as a downstream systemic effector pathway.

The differentiation, egress, and lineage origin of Peyer’s patch Tfh cells form a complete systemic relay mechanism. Teng et al. demonstrated that segmented filamentous bacteria promoted Tfh differentiation in Peyer’s patches. These cells exited Peyer’s patches in an S1PR1-dependent manner, entered systemic lymphoid tissues, and enhanced germinal-center B-cell responses, autoantibody production, and arthritis. Blocking their egress markedly attenuated disease (47). Using fate mapping, Fan et al. further showed that a subset of intestinal Th17 cells induced by segmented filamentous bacteria underwent c-Maf-dependent reprogramming into Tfh17 cells within Peyer’s patches. This aberrant Tfh population displayed enhanced migratory capacity and B-cell helper activity. Experimental expansion or depletion of these cells correspondingly aggravated or alleviated germinal-center responses and arthritis (48). Thus, intestinal Th17 cells can exploit their developmental plasticity to become Tfh17 cells that directly drive systemic autoantibody responses; intestinal immune programming can generate migratory intermediate states rather than fixed terminal phenotypes.

Fate conversion of colonic Treg cells provides another pathway linking intestinal immune history to arthritogenic activity. In PTPN2-haploinsufficient SKG mice, Hsieh et al. found that subclinical colitis markedly aggravated arthritis and promoted the accumulation of colonic-enriched RORγt+GPR15+ Treg cells in the joints. Fate and functional analyses showed that Treg cells with stronger colonic features were more likely than peripheral Treg cells to lose Foxp3 and convert into IL-17^+^ arthritogenic ex-Treg cells. PTPN2 haploinsufficiency further enhanced this conversion (49). These findings highlight that intestinal immune history can persist within migrating T cells, whose function may be reshaped after entering inflammatory tissues. Under conditions of genetic susceptibility and joint inflammation, they may instead convert into pro-inflammatory effector cells.

Once gut-associated effector cells enter the circulation, the inflamed synovial endothelium selectively recruits them through adhesion systems distinct from classical intestinal homing pathways. Salmi et al. found that human mucosal immunoblasts adhered efficiently not only to mucosal high endothelial venules but also to synovial endothelium. Synovial adhesion depended mainly on α4β1, with cooperative contributions from CD44 and LFA-1, whereas α4β7 played a limited role (50). This contrasts with intestinal homing, where α4β7–MAdCAM-1 interactions dominate, emphasizing tissue-specific trafficking programs. Subsequent studies showed that distinct mucosal leukocyte subsets use different synovial endothelial ligands. VAP-1, P-selectin, and other non-MAdCAM-1 adhesion pathways jointly support the entry of gut-associated cells into the joints (51). These findings indicate that intestinal immune experience and joint tropism can coexist within the same cell. Actual synovial entry, however, depends on joint-specific endothelial adhesion programs.

The CCR6–CCL20 axis provides an additional chemotactic mechanism for the directional accumulation of Th17 cells in inflamed joints. Hirota et al. found that arthritogenic Th17 cells preferentially expressed CCR6. Mouse and human synovial cells produced high levels of CCL20 after stimulation with IL-1β, IL-17, or TNF. CCL20 selectively attracted CCR6^+^ Th17 cells, whereas anti-CCR6 antibodies markedly reduced joint infiltration and alleviated experimental arthritis (52). Kaneko et al. observed enrichment of RORγt^+^CCR6^+^ Th17 cells in synovial fluid and synovial tissue from patients with RA. These cells displayed enhanced migration toward CCL20, providing patient-derived support for this pathway (53). In a model of enthesitis, Shi et al. further showed that blockade of CCR6–CCL20 reduced T-cell recruitment to inflamed entheses and skin and improved pathology (54). Fennen et al. found that fibroblast-like synoviocyte-derived myostatin cooperated with IL-17A to enhance CCL20 secretion and promote the transmigration of CCR6^+^ Th17 cells. Myostatin deficiency reduced joint CCL20 levels, Th17 infiltration, and chronic arthritis (55). These interactions establish a local amplification loop involving infiltrating Th17 cells, IL-17A-stimulated fibroblast-like synoviocytes, CCL20 production, and further recruitment of CCR6^+^ Th17 cells.

After entering the joint, T cells are further reshaped by synovial cytokines and matrix-derived signals into more tissue-destructive states. Using Foxp3 fate mapping, Komatsu et al. showed that IL-6 produced by joint fibroblast-like synoviocytes caused Foxp3^+^ T cells to lose their regulatory phenotype and convert into exFoxp3 Th17 cells with high IL-17 and RANKL expression. These cells displayed strong osteoclastogenic activity and directly aggravated bone erosion (56). In chronic SKG arthritis, Zoccheddu et al. further found that synovial Th17 cells converted into IL-17-negative CD44^+^ exTh17 cells. These cells exhibited greater arthritogenic activity and sustained IL-17-independent inflammation. S1P released by fibroblast-like synoviocytes promoted this conversion through S1PR4, whereas CD44 was required for the persistence of exTh17-mediated disease (57). Thus, joint injury does not simply reproduce a phenotype acquired in the intestine. Instead, it reflects further reprogramming of migrating or circulating T cells within the synovial niche.

Intestinal antigenic signals may also reach the joints through migrating myeloid cells, raising the possibility of shared antigen exposure between the intestine and joint tissues. In an SKG model of spondyloarthritis, Cai et al. found that intestinal bacterial DNA colocalized with neutrophils and pro-inflammatory macrophages. The DNA was subsequently detected in bone marrow, the spleen, and entheses. Transfer of bacterial DNA-bearing splenic or bone marrow macrophages into naive SKG mice induced more severe arthritis and expanded Th17 cells. Macrophages from germ-free SKG mice or relatively resistant BALB/c mice had substantially weaker effects (58).

Overall, the gut-joint axis illustrates how intestinal immune programming can generate migratory immune populations that are subsequently selected and reshaped within arthritic tissues. Dysbiosis, barrier disruption, and microbial metabolic signals first shape pro-inflammatory T-cell responses in the intestine. Some gut-derived T cells then migrate directly to the synovium or entheses, whereas others amplify inflammation through lymphoid organs and humoral immune pathways. After entering the joints, these cells undergo further reprogramming in response to synovial endothelial signals, chemokines, and local stromal factors. Gut-joint immune communication is therefore a multistage process involving intestinal niche-driven immune programming, cellular redistribution, and local reprogramming within the joint.

### 3.3 Gut–skin immune communication

#### 3.3.1 Intestinal imprinting of cutaneous immunity

Clinical evidence indicates that inflammatory bowel disease is associated with the acquisition of altered tissue-homing programs in circulating γδ T cells, particularly in patients with cutaneous manifestations. In patients with cutaneous manifestations, these circulating T cells display a skin-directed homing phenotype. Mann et al. compared circulating γδ T cells from healthy individuals and patients with active Crohn’s disease or ulcerative colitis. In healthy individuals, these cells commonly expressed β7 integrin but rarely expressed CCR9, cutaneous lymphocyte-associated antigen (CLA), or CCR4. CCR9 expression was markedly increased in patients with inflammatory bowel disease, whereas aberrant CLA expression was observed in those with skin manifestations (59). These findings link intestinal inflammation, altered homing programs in circulating T cells, and cutaneous involvement. They also suggest that interorgan immune communication leaves a detectable phenotypic imprint in peripheral blood.

Intestinal inflammation may also form a self-amplifying inflammatory circuit with skin barrier dysfunction. Rigoni et al. observed high expression of CLA and CCR4 on activated or memory T cells in patients with Omenn syndrome and in Rag2R229Q mice. Skin tissues simultaneously showed increased CCL17, CCL22, and broad Th1-, Th2-, and Th17-associated inflammatory signals. Further induction of colitis in this model increased the proportion of splenic CCR4+ T cells and markedly aggravated epidermal thickening, epithelial activation, and dermal Th1-cell infiltration (60). These findings indicate that persistent intestinal inflammation can amplify cutaneous T-cell inflammation in the presence of a pre-existing skin barrier defect rather than merely occurring in parallel with skin disease.

In addition to changing cellular phenotypes, microbial metabolites can serve as systemic mediators linking intestinal ecological changes to cutaneous T-cell responses. In a CARD14-driven psoriasis-like model, Wang et al. found that intestinal indole-producing bacteria increased circulating indoxyl sulfate (I3S) through host metabolic relay pathways. After entering the skin, I3S activated AHR in Th17 cells, altered chromatin accessibility, and aggravated psoriasis-like inflammation. Serum I3S levels also correlated with disease severity in patients (61). In a model of atopic dermatitis, Yu et al. further showed that intestinal epithelial TLR4 deficiency reduced *Akkermansia muciniphila*, enriched CutC-carrying bacteria, and increased circulating trimethylamine N-oxide (TMAO). TMAO directly bound PPP5, promoted PPARγ dephosphorylation, and enhanced Th2 differentiation. CD4^+^ T-cell-specific PPARγ deletion blocked this cutaneous pathological process (62). These studies establish a causal humoral basis for the effects of the gut on skin immunity. They also provide a rationale for determining whether gut-derived T cells reach the skin as cellular carriers. Importantly, these findings suggest that gut-derived signals may influence skin inflammation through both humoral pathways and cellular redistribution, raising the question of how intestinally conditioned immune cells acquire skin-selective trafficking capacity.

#### 3.3.2 Gut-derived immune-cell trafficking to the skin

The most direct evidence of gut-to-skin migration comes from local and irreversible labeling of immune cells within a defined intestinal segment. Galván-Peña et al. used colonoscopy-guided photoconversion to label only distal colonic cells in Kaede mice and tracked their systemic distribution 24 hours later. Colonic Kaede-red+ cells were almost undetectable in steady-state skin. By contrast, clearly identifiable colon-derived immune cells were present in inflamed ear skin during KLH-induced delayed-type hypersensitivity. Population analysis showed that CD4^+^ conventional T cells and γδ T cells were relatively enriched among the migrating cells, whereas B cells were rare (8). Thus, inflamed skin does not passively receive all cells leaving the colon. Instead, it selectively recruits specific T-cell subsets.

This study placed the intestinal origin, migratory direction, and cutaneous destination within a single tracing system. It directly demonstrated that inflammation can convert the skin into a new destination for colon-derived T cells. Accordingly, the central question of the gut–skin axis shifts from whether migration occurs to how intestinally conditioned immune cells acquire non-mucosal tissue tropism, how they are recruited into the skin, and how they alter local immune responses after arrival.

#### 3.3.3 Cutaneous recruitment and immune reprogramming

T cells primed in the intestine can subsequently acquire skin tropism because their homing phenotype remains reprogrammable. Mora et al. defined the dynamic nature of this process. Dendritic cells derived from mesenteric lymph nodes and Peyer’s patches induced CD8^+^ T cells to express α4β7 and CCR9 and enhanced their entry into the small intestine. By contrast, dendritic cells from skin-draining peripheral lymph nodes mainly induced E- and P-selectin ligands, enabling more efficient adhesion to cutaneous vessels and entry into inflamed skin (63). Importantly, when effector-memory T cells with established gut- or skin-homing phenotypes encountered antigen again in the presence of the other dendritic-cell population, their original homing program was progressively revised toward the tissue tropism associated with the most recent antigen-presenting environment (63). Dudda et al. similarly showed that dendritic-cell origin, soluble factors, and subsequent tissue environments can reprogram previously established tissue-selective homing patterns (64). Thus, T cells initially directed toward the intestine are not permanently confined to this organ. They may acquire the capacity for cutaneous recruitment in a new antigen-presenting environment. Unlike intestinal homing, which relies largely on α4β7-associated mucosal recruitment, skin localization requires a distinct combination of selectin ligands, chemokine receptors, and integrins.

The inflamed intestine may generate pro-inflammatory T cells with both mucosal- and skin-homing potential. In a human ex vivo system, Gordon et al. found that intestinal dendritic cells induced not only α4β7 but also CLA, α4β1, and FUT7, thereby generating CLA^+^α4β7^+^ dual-homing CD4^+^ T cells. IL-12 and IL-23 enhanced this phenotype. These cells were increased in intestinal tissues from patients with Crohn’s disease and were enriched for the production of IFN-γ, IL-17, and TNF (65). Consistent with these findings, Hoffmann et al. observed coexpression of P-selectin ligand and α4β7 on mesenteric lymph node CD4^+^ T cells during experimental colitis. This finding indicates that intestinal inflammation can impose both mucosal- and skin-recruitment programs on the same cell (66).

Selectins, integrins, and chemokines in the cutaneous microvasculature determine whether circulating T cells ultimately enter inflamed skin. Tietz et al. demonstrated that CD4^+^ T cells lacking E- and P-selectin ligands were almost completely unable to enter inflamed skin. This finding established selectin-dependent capture and rolling as essential initial steps in cutaneous recruitment (67). Campbell et al. further found that CCR4 deficiency did not prevent the formation of circulating E-selectin ligand-positive cells but markedly reduced their antigen-driven accumulation in the skin. CCR4 therefore provides a critical chemotactic positioning signal after selectin-mediated rolling (68). Gehad et al. showed that memory CD4^+^ T cells and recently activated T cells differ in their dependence on CCR4, E-selectin, and α4β1. These findings suggest that inflamed skin can select T cells at different differentiation states through distinct combinations of adhesion pathways (69).

Reappearance of cognate antigen in the skin can convert homing plasticity into actual cutaneous recruitment and inflammation. This process has been functionally validated in gut-to-skin migration models. Oyoshi et al. first induced α4β7^+^CCR4−E-selectin-ligand− gut-homing-like CD4^+^ T cells by oral administration of OVA with cholera toxin and then rechallenged the epidermis with OVA. Antigen-specific cells acquired CCR4 and E-selectin ligands in lymph nodes, migrated into the skin, and induced epidermal thickening, eosinophil and CD4^+^ T-cell infiltration, and increased Th2-associated mediators. Mesenteric lymph node α4β7+ T cells transferred the dermatitis, whereas CCR4 deficiency or the absence of lymph nodes markedly reduced cutaneous recruitment and pathology (70). Comparative experiments by Glocova et al. further showed that both gut-primed and skin-primed OVA-specific T cells could reach skin-draining lymph nodes after epidermal antigen rechallenge. However, only skin-primed cells induced substantial IL-22/IL-17A-associated dermatitis. Thus, arrival in a skin-associated compartment and acquisition of a cutaneous pathogenic program are sequential but distinct events (71).

Intestinally primed T cells may also enter the skin through S1P-dependent egress and induce long-term changes in local antigen responses. Classon et al. used a *Heligmosomoides polygyrus* infection strictly restricted to the intestine. They observed a two- to threefold increase in cutaneous CD4^+^ T cells, which were predominantly IL-4^+^IL-13^+^ Th2 cells reactive to helminth antigens. These cells upregulated CCR4 and CCR10 in mesenteric lymph nodes and blood. Their accumulation in the skin was not attributable to local proliferation. Blocking lymphocyte egress with FTY720 during infection abolished both cutaneous Th2 accumulation and helminth antigen-specific skin responses. The population persisted after deworming and suppressed Th1 recall responses to an unrelated mycobacterial antigen (72). These findings indicate that intestinal infection can durably reshape cutaneous immune memory through T-cell migration.

Migration of gut-derived T cells may also disrupt established tolerance to skin commensals. Merana et al. first established *Staphylococcus epidermidis*-specific cutaneous Treg responses during the neonatal period and then induced DSS- or *Il10*-deficiency-associated colitis. Colitis reduced the proportion of commensal-specific Treg cells and increased effector CD4^+^ T cells in both gut- and skin-draining lymph nodes. These changes were accompanied by neutrophil infiltration in the skin. KikGR tracing and circulation-blocking experiments showed that colitis increased CD4^+^ T-cell traffic between gut- and skin-draining lymph nodes. T-cell-intrinsic IL-1R1 deficiency preserved the dominance of commensal-specific Treg cells and reduced cutaneous immune imbalance (73). This study established a continuous mechanistic sequence involving intestinal inflammation, intercompartmental lymphocyte traffic, IL-1R1-dependent effector skewing, and loss of cutaneous tolerance.

Overall, gut-skin immune communication represents a sequential process in which intestinal conditioning establishes migratory potential, skin-selective recruitment determines tissue entry, and local antigenic and cytokine environments define pathogenic outcomes. Intestinal antigen presentation and inflammation generate T cells with plastic homing programs, reflecting the capacity of intestinal immune niches to imprint transferable tissue-recognition states. These cells can be reprogrammed by skin-derived soluble factors and acquire a cutaneous-homing phenotype. Inflamed skin then selectively recruits them through selectins, CCR4/CCR10, and integrin pathways. Local antigens and cytokines ultimately determine whether the resulting response manifests as allergic inflammation, loss of tolerance, or long-term immune remodeling.

### 3.4 Gut–brain immune communication

#### 3.4.1 Immune imprinting linking intestinal perturbation to CNS inflammation

The intestinal microbiota was among the first factors shown to shape the generation of encephalitogenic immune programs, providing early evidence that intestinal immune conditioning can influence CNS inflammation. In experimental autoimmune encephalomyelitis (EAE), Lee et al. found that germ-free mice had markedly reduced intestinal Th17 cells and showed parallel reductions in disease incidence and severity. Recolonization with segmented filamentous bacteria restored intestinal Th17 responses and susceptibility to EAE (74). In a spontaneous demyelination model, Berer et al. further showed that myelin-reactive T cells were already present but required the conventional microbiota for full activation and induction of central nervous system (CNS) autoimmunity (75). Miyauchi et al. subsequently used a defined microbial community to separate this process into two cooperative components. One bacterial group provided antigenic stimuli capable of activating myelin-reactive T-cell receptors, whereas another induced a serum amyloid A/IL-23-associated environment that expanded Th17 responses. Spinal cord inflammation was most severe when both signals were present (76).

Intestinal immune dysregulation can also alter the inflammatory outcome of non-autoimmune brain injury. Singh et al. transferred post-stroke dysbiotic microbiota into recipient mice. These mice developed stronger pro-inflammatory peripheral immune responses and more severe neuroinflammation and tissue injury after cerebral ischemia, indicating that stroke-associated microbial alterations exert transferable pathogenic effects (77). In a model of chronic colitis, Mickael et al. observed that the number of brain-infiltrating CD4^+^ T cells increased with colitis severity and was accompanied by hypothalamic astrocyte activation and neurobehavioral abnormalities. Both the brain-infiltrating cells and pathogenic colonic Th17 cells expressed RORγt. Transfer of RORγt-deficient CD4^+^ T cells markedly reduced brain infiltration and behavioral abnormalities (78). These findings link persistent intestinal inflammation to the generation and redistribution of inflammatory T-cell responses toward the CNS.

The effects of the intestinal microbiota on CNS inflammation are not uniformly pro-inflammatory. Rather, they depend on the immune programs established within the intestinal immune niche. Mangalam et al. isolated *Prevotella histicola* from the intestine of a healthy individual and colonized EAE mice with this bacterium. Treated mice showed reduced Th1/Th17 responses and fewer inflammatory dendritic cells, together with increased Treg cells, tolerogenic dendritic cells, and suppressive macrophages. These changes ultimately reduced demyelination and clinical scores (79). Thus, the intestinal microbiota can either establish an encephalitogenic immune background or limit CNS inflammation by enhancing immune tolerance.

These studies establish robust associations among the intestinal microbiota, mucosal T-cell states, and CNS inflammation. Demonstrating that immune cells themselves transmit this connection requires spatial labeling of intestinal cells and subsequent tracking of their destinations within the CNS.

#### 3.4.2 Evidence for gut-derived immune-cell trafficking to the CNS

Local intestinal photoconversion provided direct evidence that intestinal immune cells can traffic to the leptomeninges after cerebral ischemic injury. Benakis et al. locally photoconverted the distal small intestine of KikGR mice, permanently changing the cells present in that segment from green to red. After stroke, red-labeled T cells were markedly increased in the leptomeninges. Antibiotic-mediated microbiota remodeling increased small-intestinal Treg cells and IL-10, reduced IL-17-producing γδ T cells, and decreased both photoconverted effector T cells in the meninges and brain injury. This experimental system therefore linked microbial changes, intestinal T-cell composition, cellular migration, and stroke outcome (80).

Brea et al. subsequently used KikGR photoconversion for comprehensive tracking of small-intestinal immune cells and showed that stroke broadly promotes their systemic egress. After photoconversion, red-labeled cells were examined in peripheral lymphoid organs, the meninges, and brain tissue. Compared with sham-operated animals, mice examined 3 days after stroke showed marked increases in gut-derived cells across several tissues. Large numbers of CD45^hi^KikR^+^ cells were detected in the brain and meninges, with particularly prominent migration of CD11c^+^ dendritic cells and macrophages (81). This study extended the analysis beyond T cells to mixed intestinal immune-cell populations and demonstrated that stroke induces broad mobilization of gut-derived immune cells rather than changes in a single lymphocyte subset.

Chronic intestinal inflammation can further increase the influx of gut-derived CD4^+^ T cells into the meninges. Feng et al. combined chronic DSS colitis with a photothrombotic stroke model and traced intestinally derived cells. On day 7 after stroke, mice with colitis showed a marked increase in gut-derived CD4^+^ T cells in the meninges. They also displayed greater peripheral CD4^+^ T-cell infiltration in the peri-infarct region, increased M1-like microglia/macrophages, and enhanced neuronal loss. Selective depletion of meningeal macrophages reduced both parenchymal T-cell infiltration and infarct volume. These findings suggest that the meninges may serve as an intermediate amplification niche where gut-derived CD4^+^ T cells interact with local myeloid cells to propagate cerebral inflammation (82).

#### 3.4.3 Recruitment and reactivation of gut-derived immune cells in the CNS

Antigen presentation by intestinal epithelial cells can promote the local priming of encephalitogenic CD4^+^ T cells. Suzuki et al. found that intestinal epithelial antigen-presentation pathways were upregulated in both EAE mice and patients with multiple sclerosis. Ileal epithelial MHC-II expression was particularly prominent during EAE. Intestinal epithelial-specific deletion of MHC-II markedly reduced both the generation of pathogenic intestinal Th17 cells and EAE severity. In organoid cocultures, intestinal epithelial organoids expressing cognate myelin oligodendrocyte glycoprotein antigen induced RORγt^+^CD4^+^ T cells in an MHC-II-dependent manner. Parabiosis and local photoconversion further confirmed that these Th17 cells, generated in the intestinal lamina propria, subsequently entered the spinal cord (83). This study established a continuous pathway from intestinal epithelial presentation of self-antigen to local Th17 generation, spinal cord migration, and EAE.

Microbial metabolites and antigen uptake in Peyer’s patches represent two additional pathways of local immune programming. Miyamoto et al. found that microbiota-derived kynurenic acid (KYNA) recruited Ly6C^+^ monocytes/macrophages into the small intestine through GPR35 and induced pro-inflammatory signals such as IL-6 and TNF. GPR35-dependent myeloid-cell accumulation expanded the intestinal Th17 environment and aggravated experimental encephalitis (84). Komiyama et al. localized the generation of encephalitogenic γδT17 cells to Peyer’s patches. After M cells captured specific lactobacilli, bacterial antigens stimulated the T-cell receptors of CXCR6^hi^Vγ6^+^Vδ1^+^ γδT17 cells and induced an encephalitogenic phenotype. Impairing M-cell function reduced EAE severity, whereas increasing M-cell abundance aggravated disease. Activated γδT17 cells subsequently entered the CNS during early EAE and initiated type 17 inflammation (85). These studies show that metabolite-driven myeloid licensing and M-cell-mediated uptake of commensal antigens can both shape effector T cells within the intestine.

The entry of intestinally conditioned effector T cells into the CNS is coordinated by chemokine-guided positioning signals. Reboldi et al. found that CCR6-deficient mice generated peripheral Th17 responses but failed to efficiently initiate EAE. CCR6-expressing Th17 cells entered the cerebrospinal fluid compartment along a CCL20 gradient generated by the choroid plexus epithelium. Transfer of these cells restored disease (86).

At cerebral and spinal microvessels, integrins and endothelial adhesion molecules convert circulating effector T cells from transiently interacting cells into firmly adherent and transmigrating cells. Using intravital imaging of spinal cord microcirculation, Vajkoczy et al. found that the interaction between α4 integrin and VCAM-1 rapidly captured encephalitogenic T cells and stabilized their arrest on white-matter microvessels without requiring G protein signaling. Blocking this interaction markedly reduced both cellular adhesion and tissue entry (87). Charabati et al. combined single-cell transcriptomics of cerebral endothelial cells, *in vitro* barrier models, and functional blockade. They found that MCAM^+^ brain endothelial cells displayed a stronger inflammatory recruitment phenotype and selectively promoted the passage of Th1/Th17 cells across the blood–brain barrier. MCAM inhibition reduced both T-cell migration and neuroinflammation (88). These results indicate that inflamed CNS endothelium actively establishes an adhesive interface that facilitates effector T-cell entry.

Migrating cells must receive local antigenic restimulation in the meninges before transient arrival can develop into sustained tissue inflammation. Using two-photon intravital imaging, Bartholomäus et al. observed that encephalitogenic T cells initially arrested and crawled within leptomeningeal vessels. After crossing the endothelium, they scanned the perivascular space and formed stable contacts with phagocytes presenting myelin antigens. These interactions triggered the release of pro-inflammatory mediators and preceded further invasion of the brain parenchyma (89). In MOG-EAE, Kivisäkk et al. similarly found that Th1/Th17-like CD4^+^ T cells accumulated in the subarachnoid space before appearing in the spinal cord parenchyma. These cells maintained prolonged contacts with MHC-II^+^ antigen-presenting cells and underwent local proliferation (90). Thus, the meninges serve not only as a transit site but also as an immune amplification compartment in which infiltrating T cells regain effector competence.

Th17 cells with gut-associated and encephalitogenic clonal histories may converge in the spleen, where they undergo further functional diversification. Using IL-17 fate mapping, single-cell transcriptomics, and T-cell receptor clonotype analysis, Schnell et al. identified a homeostatic TCF1^+^SLAMF6^+^IL-17^+^ stem-like Th17 population and an encephalitogenic GM-CSF^+^IFN-γ^+^CXCR6^+^ population that expanded during EAE. Antibiotic-mediated microbiota depletion caused an approximately ninefold reduction in fate-mapped Th17 cells across tissues, accompanied by a proportional decrease in splenic SLAMF6^+^ cells. Antibiotic-treated mice were also resistant to EAE and showed marked reductions in both SLAMF6^+^ and CXCR6^+^ populations, consistent with microbiota-dependent maintenance of the stem-like reservoir and reduced generation of downstream pathogenic effectors. Moreover, selective photoconversion of intestinal tissues in Kaede mice resulted in the detection of photoconverted CXCR6^+^CD4^+^ T cells in the spleen and CNS, where these cells produced IFN-γ and GM-CSF (91). Thus, this study supports the spleen as a systemic relay containing both gut-associated and encephalitogenic Th17 populations, but it does not conclusively establish that the pathogenic stem-like reservoir was initially primed in the intestine.

Antigen cross-reactivity provides a further explanation for how T cells specific for intestinal microorganisms may be reactivated within the CNS. White et al. established a system of segmented filamentous bacterium-specific CD4^+^ T cells and found that intestinal inflammation enabled these cells to acquire CNS-infiltrating capacity. After entering the CNS, some clones recognized antigens derived from host proteins, including ERBB2, TRO, and ANAPC2. They subsequently produced large amounts of GM-CSF, IFN-γ, and IL-17A and activated microglia through IL-23R-associated programs and GM-CSF (92). Ma et al. further showed that microbiome-derived peptides mimicking MOG could activate MOG-specific CD4^+^ T cells and alter the course of EAE (93). In patients with multiple sclerosis, Planas et al. identified CD4^+^ T-cell clones from cerebrospinal fluid and brain-associated compartments that reacted to GDP-L-fucose synthase and homologous microbial peptides. These findings suggest that molecular mimicry may also operate in human disease (94).

The effects of gut-derived T cells are further determined by the balance between regulatory and pro-inflammatory programs. Wang et al. found that the commensal-derived polysaccharide A induced CD39^+^CD4^+^ Treg cells and enhanced their migratory phenotype in CNS-draining lymphoid compartments. CD39-mediated purinergic regulation promoted Treg migration and suppressed neuroinflammation in EAE (95). Thus, intestinal T-cell output to the CNS does not represent a uniformly pro-inflammatory signal. Its direction depends on cell lineage, migratory capacity, and local reactivation.

In summary, gut–brain T-cell communication can be organized into a continuous three-step mechanism involving intestinal programming, systemic trafficking, and CNS reactivation. First, the intestinal microbiota, epithelium, and mucosal immune cells determine antigen selection and functional programming. Second, T cells that acquire egress competence enter the circulation and are recruited to the CNS through the choroid plexus, meninges, or cerebral microvasculature. Third, these cells receive local antigen-presentation, cytokine, and tissue-retention signals after arrival, ultimately producing neuroinflammatory, tissue-damaging, or homeostatic regulatory effects. The trafficking routes of gut-derived immune cells to distant organs are schematically summarized in **Figure 2**.

**Figure 2:**
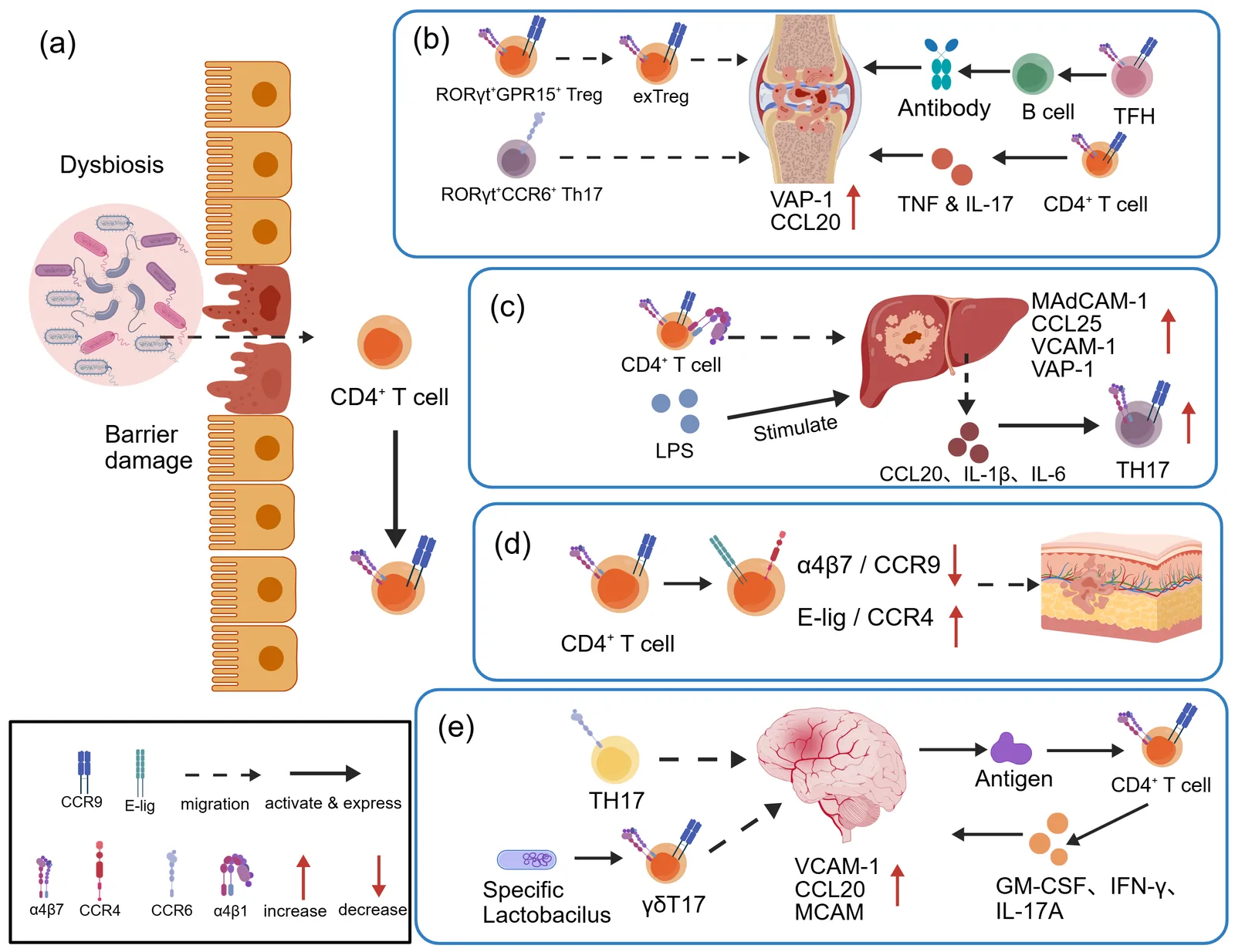
Aberrant redistribution and tissue-specific reprogramming of gut-programmed immune cells across gut–organ axes. **(a)** Intestinal dysbiosis and epithelial barrier damage disrupt mucosal immune homeostasis and facilitate the generation and systemic dissemination of gut-programmed CD4⁺ T cells. **(b)** In the gut–joint axis, intestinally programmed T-cell populations contribute to arthritis through complementary cellular and humoral routes. Colonic-enriched RORγt⁺GPR15⁺ Treg-lineage cells can accumulate in the joints and undergo Foxp3 loss and conversion into arthritogenic ex-Treg cells, whereas RORγt⁺CCR6⁺ Th17 cells are preferentially recruited to inflamed joint tissues through the CCR6–CCL20 axis. Gut-derived effector T cells can retain TNF and IL-17 production, while intestinal Tfh/Tfh17 cells promote germinal-center B-cell responses and autoantibody production. Joint-specific endothelial adhesion and chemokine programs further support cellular recruitment and local inflammatory amplification. **(c)** In the gut–liver axis, inflamed hepatic vasculature acquires mucosal-like recruitment features, including MAdCAM-1 and CCL25 expression, which facilitate the recruitment of α4β7⁺ and CCR9⁺ lymphocytes, whereas VCAM-1–α4β1 interactions support intrahepatic retention. Microbial products such as LPS, together with Th1/Th17-associated inflammatory signals, stimulate biliary epithelial cells to produce CCL20, IL-1β, and IL-6, thereby promoting Th17-cell chemotaxis, maintenance, and local expansion. **(d)** In the gut–skin axis, tissue-homing programs remain plastic after intestinal priming. Gut-homing T cells can be redirected from an α4β7/CCR9-associated mucosal program toward an E-selectin ligand/CCR4-associated cutaneous program, enabling their recruitment to inflamed skin following tissue-specific antigenic and inflammatory signals. **(e)** In the gut–brain axis, microbiota-conditioned Th17 cells and γδT17 cells induced by specific lactobacilli can acquire CNS migratory capacity. Their recruitment is facilitated by CCL20–CCR6, VCAM-1–α4-integrin, and MCAM-associated endothelial pathways. Following entry into CNS-associated compartments, local antigenic restimulation promotes effector responses, including the production of GM-CSF, IFN-γ, and IL-17A by CD4⁺ T cells, thereby amplifying neuroinflammation. Collectively, dysbiosis, barrier injury, and intestinal immune imbalance promote the generation of gut-derived lymphocytes with inflammatory or plastic phenotypes. After entering the circulation, these cells migrate to the liver, joints, skin, and brain under the guidance of organ-specific adhesion molecules, chemokines, and antigenic signals, where local tissue environments retain or reactivate them and amplify extraintestinal inflammation. Created with BioGDP.com (127).

### 3.5 Immune migration to other organs

#### 3.5.1 Gut–heart immune communication

Current evidence supports intestinal immune programming as a contributor to cardiac inflammation, although direct *in situ* evidence for gut-derived T-cell trafficking to the heart remains limited. Gil-Cruz et al. found that molecular-mimicry peptides derived from commensal *Bacteroides* species activated cardiac myosin-specific Th17 cells in the intestine. These cells subsequently promoted the progression of autoimmune myocarditis to lethal inflammatory cardiomyopathy. This study established a causal link between intestinal antigenic programming, heart-reactive T-cell generation, and myocardial injury, but did not determine whether these cells physically migrate from the intestine to the heart (96). Tian et al. isolated ILCs from the intestinal lamina propria of donor mice and adoptively transferred them into recipients with viral myocarditis. Donor-derived ILC3s were subsequently detected in the heart. S1PR1 inhibition reduced cardiac ILC3 accumulation, whereas intestinal ILCs upregulated CXCR6 and showed concurrent increases in CXCL16. These findings suggest that gut-derived ILC3s may leave the intestine in an S1PR1-dependent manner and be recruited to the inflamed heart through the CXCR6–CXCL16 axis (97). However, because this study used adoptive transfer rather than *in situ* intestinal fate mapping, it primarily supports cellular origin and migratory capacity. Stoffers et al. further demonstrated that GPR15–GPR15L mediates the entry of Treg and cytotoxic T cells into virus-infected cardiac tissue and promotes viral clearance, but did not establish an intestinal origin for these cells (98). At present, gut–heart immune communication is therefore best summarized as follows: intestinal microbes first shape heart-reactive lymphocytes, and the inflamed heart subsequently recruits circulating cells through specific homing pathways. Whether intestinally programmed T cells directly traffic to the heart under physiological disease conditions remains an important unresolved question requiring *in situ* fate-mapping approaches.

#### 3.5.2 Gut–kidney immune communication

Among additional organ axes, the gut-kidney axis provides some of the strongest evidence for gut-derived immune-cell trafficking. Krebs et al. locally photoconverted the small intestine of Kaede mice and detected gut-derived Kaede-red^+^ CD4^+^ T cells in kidneys affected by crescentic glomerulonephritis. The migrating population showed enrichment of Th17 cells, consistent with intestinal programming of inflammatory effector phenotypes. Gut-derived Th17 cells exited the small intestine through S1PR1-dependent mechanisms and were subsequently recruited from the circulation by the CCR6–CCL20 axis in the inflamed kidney. Germ-free conditions or antibiotic treatment reduced intestinal Th17 cells and alleviated nephritis, whereas infection with *Citrobacter rodentium* expanded intestinal Th17 cells and aggravated disease (99). Importantly, CCR6-CCL20 signaling is not intrinsically pathogenic, as it can recruit both inflammatory Th17 cells and regulatory Treg cells. Its net effect therefore depends on the relative proportions of pro-inflammatory and regulatory cells entering the kidney (100). Liang et al. further demonstrated that gut-derived CXCR6^+^ ILC3s migrated from the intestinal mucosa into fibrotic kidneys, where injured renal tubules released CXCL16 to direct their recruitment. After entering the kidney, ILC3s upregulated PD-1. PD-1 inhibited IL-23R internalization, thereby enhancing JAK2–STAT3–RORγt signaling and IL-17A production. This response activated renal fibroblasts and promoted extracellular matrix deposition (101). Together, these findings reveal distinct cellular routes through which intestinal immune programs are transferred to the kidney. The S1PR1–CCR6/CCL20 pathway enables gut-derived Th17 cells to enter inflamed kidneys and aggravate glomerulonephritis, whereas CXCR6–CXCL16 recruits gut-derived ILC3s to injured kidneys, where the PD-1–IL-23R–IL-17A pathway promotes fibrosis.

#### 3.5.3 Gut–lung immune communication

The gut-lung axis provides evidence that intestinal immune-cell trafficking extends beyond adaptive T cells to include rapidly mobilized innate lymphoid populations. Xie et al. selectively photoconverted the small intestine of Kaede mice and detected gut-derived memory-like γδ T cells in the lungs during sepsis. These cells served as an early source of pulmonary IL-17A and aggravated acute lung injury through neutrophilic inflammation. Wnt/β-catenin signaling in alveolar macrophages induced CCL1 expression and established a local chemotactic environment for the recruitment of gut-derived γδ T cells into the lung (102).

A second migratory route is mediated by ILC2s. Huang et al. first demonstrated that IL-25 stimulation or helminth infection mobilized inflammatory ILC2s from the intestine. These cells exited the gut through an S1P-dependent mechanism and entered the lungs (103). Can et al. subsequently used *in situ* intestinal photoconversion to confirm that circulating ILC2s entering the lungs during early infection were derived mainly from the intestine, whereas bone marrow progenitors primarily replenished resident pulmonary ILC2s (104). Ito et al. further resolved this process into sequential steps. S1PR5 controlled ILC2 movement from intestinal tissues into lymphatic vessels, whereas S1PR1 mediated their exit from mesenteric lymph nodes into the blood and subsequent arrival in the lungs (105). Thus, intestinal immune export can produce divergent outcomes in the lung, with gut-derived γδ T cells promoting inflammatory injury and intestinal ILC2s supporting type 2 immunity and tissue repair. These findings indicate that the intestine influences lung immunity not only through circulating metabolites but also through the direct export of lymphocytes with defined effector functions.

## 4 Therapeutic strategies targeting gut-derived immune redistribution

### 4.1 Blocking downstream trafficking

Integrins and adhesion molecules represent the final checkpoint of immune-cell tissue entry and therefore provide the most direct opportunity to interfere with immune-cell trafficking. Natalizumab has demonstrated efficacy in both relapsing multiple sclerosis and Crohn’s disease, indicating that targeting shared trafficking pathways can influence inflammatory processes across distinct organs (106, 107). However, the risk of natalizumab-associated progressive multifocal leukoencephalopathy is influenced by anti-JC virus antibody status, prior immunosuppressant exposure, and treatment duration, highlighting the challenge of immune surveillance (108). Vedolizumab selectively blocks α4β7. A recent meta-analysis showed improvement in approximately 40% of pre-existing extraintestinal manifestations, although worsening and *de novo* manifestations were also reported. These findings suggest that distal benefits may occur when extraintestinal manifestations share inflammatory dependence with intestinal disease activity; such benefits should not be interpreted as direct treatment of independent articular, cutaneous, or ocular disease (109). Long-term safety data further support a favorable safety profile for vedolizumab during extended treatment (110).

Accordingly, the focus of drug development has shifted from whether adhesion can be inhibited to how tissue selectivity, ease of administration, and long-term safety can be optimized. The oral α4 antagonist carotegrast methyl improved induction response in a phase 3 trial of ulcerative colitis, supporting the feasibility of small-molecule anti-adhesion therapy; however, its cross-organ therapeutic value and long-term systemic risks remain to be established (111). By contrast, etrolizumab, which targets processes involving both α4β7 and αEβ7, failed to demonstrate superiority over infliximab in the phase 3 GARDENIA trial, indicating that broader target coverage does not necessarily translate into more consistent clinical benefit (112). Future development should therefore prioritize mechanistically informed patient stratification based on target expression, affected organs, and disease stage, together with tissue-selective delivery and long-term immune monitoring.

Beyond adhesion molecules, chemokine networks provide directional signals that determine where circulating immune cells ultimately accumulate. However, translating individual chemokine blockade into clinical benefit has been challenging. The CCR9 antagonist vercirnon failed to improve the primary clinical endpoint in a phase 3 trial of Crohn’s disease, and the dual CCR2/CCR5 antagonist cenicriviroc did not improve NASH-related liver fibrosis in the phase 3 AURORA trial (113, 114).

These observations suggest that when trafficking pathways are redundant, targeting downstream effector programs may provide broader therapeutic control. Shared inflammatory pathways such as TNF, IL-12/23, and IL-23 lie at effector nodes used by multiple cell populations and organs and have achieved more consistent clinical success. Infliximab and ustekinumab established the efficacy of TNF and IL-12/23 blockade, respectively, in Crohn’s disease, while the recent phase 3 VIVID-1 trial showed that the IL-23p19 inhibitor mirikizumab can effectively induce and maintain remission (115–117). Collectively, these findings suggest that shared inflammatory effector pathways may represent more robust therapeutic nodes than individual homing axes.

Modulating immune-cell states may provide a broader strategy by simultaneously reshaping cytokine production, effector differentiation, and migration potential. The induction and maintenance efficacy of tofacitinib in ulcerative colitis, together with positive phase 3 results for upadacitinib in both ulcerative colitis and Crohn’s disease, have established JAK signaling as one of the best-supported oral therapeutic nodes in inflammatory bowel disease (118–120). These agents have a rapid onset of action and broad immunomodulatory activity, but treatment requires attention to serious infections and herpes zoster. Cardiovascular and malignancy risks identified with tofacitinib in a cardiovascular-risk-enriched rheumatoid arthritis population further support risk-based patient selection for JAK inhibition (121). These findings emphasize the importance of risk-adapted patient selection when targeting broadly acting immune-regulatory pathways.

### 4.2 Modulating upstream intestinal immune programming

Because intestinal immune programming originates upstream of immune-cell trafficking, microbiome-based interventions represent a theoretically attractive approach to reshape multiple layers of the gut–organ communication network. Microbiome modulation operates upstream of the immune-trafficking cascade and could theoretically influence intestinal barrier function, antigenic stimulation, T-cell differentiation, and microbial metabolites simultaneously, thereby linking the gut to distant organs. Current evidence nevertheless indicates that whole-ecosystem manipulation remains difficult to standardize and predict. In a randomized trial of active peripheral psoriatic arthritis, donor fecal microbiota transplantation (FMT) did not improve outcomes and was associated with a higher rate of treatment failure (122). A small randomized pilot study in multiple sclerosis primarily established tolerability and showed that intestinal permeability and microbial community structure could be modified (123). The open-label EXPLORER study in systemic lupus erythematosus reported signals of safety and reduced disease activity (124), but evidence remains insufficient to establish reproducible clinical efficacy.

Compared with donor FMT, defined microbial preparations with controlled composition and dosing may provide greater translational feasibility. The gut-restricted, non-live preparation EDP1815 showed a safety profile comparable to placebo and a modest cutaneous efficacy signal in a phase 2 psoriasis trial, suggesting that intestinally restricted microbial-derived signals may induce systemic immune modulation without systemic microbial exposure (125). The SER-287 study further highlighted that the efficacy of defined microbial consortia depends strongly on ecological conditions, including pretreatment and dosing frequency (126). In animal models, Prevotella histicola suppressed Th1/Th17 responses, enhanced regulatory immunity, and attenuated central nervous system inflammation. These findings provide mechanistic support for the concept that microbiota-driven immune reprogramming in the intestine can influence distal inflammatory outcomes (79). At present, microbiome-based interventions are more likely to complement rather than replace organ-directed therapies, particularly by modifying upstream immune programming and altering the threshold for distal inflammatory activation.

## 5 Conclusion

Increasing evidence supports the concept that the gut functions as a systemic immune regulatory hub rather than an isolated mucosal compartment, continuously integrating microbial, dietary antigenic, and host-derived signals to shape immune-cell function, differentiation, and migratory potential. Through soluble mediator dissemination, microbial or antigenic translocation, and immune-cell trafficking, intestinal perturbations can influence inflammatory states in distant tissues.

However, the egress of gut-derived immune cells does not necessarily result in organ damage. The biological consequences of such migration depend on an integrated set of factors, including cellular lineage and antigen specificity, homing-receptor expression, endothelial recruitment programs, and local processes such as antigen presentation, tissue retention, and phenotypic reprogramming. Effector T cells may amplify inflammation, whereas regulatory T cells and certain innate lymphoid-cell subsets may exert protective effects. These observations underscore the strong tissue-context and disease-stage dependence of gut–organ immune communication. Accordingly, studies of gut-organ immune communication should distinguish association from causal trafficking by defining cellular origin, migratory direction, and functional contribution. Microbial dysbiosis or intertissue clonal similarity alone should not be considered sufficient evidence for gut-derived immune-cell migration.

From a therapeutic perspective, targeting shared cytokine pathways and JAK signalling has already demonstrated the potential to achieve effects across organ-specific inflammatory diseases. Interventions based on microbiota modulation, defined microbial strains, or microbial metabolites may provide opportunities to intervene earlier at the stage of intestinal immune programming. Nevertheless, their clinical translation remains constrained by heterogeneous treatment responses, redundancy within chemokine networks, and insufficient patient stratification. Future studies should define the mechanistic chain linking tissue origin, immune-cell migration, and distal-organ outcomes. Such an approach will be essential for identifying cross-organ immune targets with causal relevance and developing more precise therapeutic strategies.
